# Impact of perioperative glucocorticoids on glycemic control, PONV, and acute pain after primary bilateral total knee arthroplasty: a systematic review and meta-analysis of randomized controlled trials

**DOI:** 10.1186/s13018-025-06521-5

**Published:** 2025-12-05

**Authors:** Omar Abdelaziz, Ziad G. Zayed, Mohamed Abdo Khalafallah, Ahmed Mohamed Elsayed, Mohamed A. Hanafi

**Affiliations:** 1 Faculty of Medicine, Alexandria University, Alexandria, Egypt; 2 Faculty of Medicine, Al-Azhar University, New Damietta, Egypt; 3 College of Human Medicine, Benha University, Benha, Egypt

**Keywords:** Bilateral total knee arthroplasty, Perioperative glucocorticoids, Postoperative pain, PONV, Glycemic control, ERAS

## Abstract

**Background:**

Perioperative glucocorticoids have been increasingly used in total knee arthroplasty (TKA) to optimize postoperative recovery, with prior evidence suggesting potential reductions in postoperative nausea and vomiting (PONV) in unilateral TKA. However, their safety and efficacy in primary bilateral total knee arthroplasty (BTKA) remain uncertain, particularly regarding pain relief, glycemic control, and PONV, given the greater surgical stress and limited BTKA-specific data. This systematic review and meta-analysis aimed to evaluate the impact of perioperative glucocorticoids on these outcomes in patients undergoing primary BTKA.

**Methods:**

Following the Preferred Reporting Items for Systematic Reviews and Meta-Analyses (PRISMA) and Cochrane guidelines, a comprehensive search of PubMed, Web of Science, and Scopus databases was conducted up to August 13, 2025. Randomized controlled trials (RCTs) comparing perioperative glucocorticoids with placebo or standard care in adult patients undergoing primary bilateral TKA were included. Data were pooled using a random-effects model, and results were expressed as mean differences (MDs) or risk ratios (RRs) with 95% confidence intervals (CIs). The protocol was registered in PROSPERO (CRD420251152310). Primary time points were POD1–3 for pain and PACU/POD1 for glycemia; hierarchy prioritized pain with movement over rest and peak glucose values over daily means.

**Results:**

Five RCTs encompassing 245 patients were included. Glucocorticoid administration led to a statistically significant but modest reduction in early postoperative pain at rest on day 1 (MD ≈ − 0.46 on VAS, 95% CI − 0.80 to − 0.12; *p* = 0.01) with limited clinical relevance, consistent across IV and local routes (*p* = 0.73 for subgroup difference) and during activity (pooled MD = − 1.06, 95% CI − 1.64 to − 0.49; *p* < 0.001). Opioid consumption showed no significant reduction (MD = − 24.63 mg, 95% CI − 99.25 to 50.00; *p* = 0.52). Intravenous glucocorticoids were associated with a transient rise in postoperative glucose levels (pooled MD = 22.19 mg/dL, 95% CI 12.35 to 32.02; *p* < 0.001), but without a significant increase in wound complications (RR = 1.14, 95% CI 0.41 to 3.17; *p* = 0.80) or infection rates (RR = 1.00, 95% CI 0.18 to 5.69; *p* = 1.00). Glucocorticoid use had no significant effect on PONV (nausea: RR = 1.00, 95% CI 0.48 to 2.07, *p* = 1.00; I^2^ = 0%; vomiting: RR = 0.96, 95% CI 0.65 to 1.42, *p* = 0.84; I^2^ = 0%), functional recovery, or hospital stay duration.

**Conclusion:**

Perioperative glucocorticoids appear to reduce early postoperative pain in bilateral total knee arthroplasty (BTKA), particularly when combined with local infiltration analgesia (LIA), without increasing complications. However, despite prior evidence suggesting PONV reduction in unilateral TKA, no significant effect was observed on PONV, opioid use, or functional outcomes in BTKA, and transient hyperglycemia may occur with intravenous administration. Further high-quality BTKA-specific randomized controlled trials are warranted before glucocorticoids can be considered a standard component of Enhanced Recovery After Surgery (ERAS) protocols.

**Supplementary Information:**

The online version contains supplementary material available at 10.1186/s13018-025-06521-5.

## Introduction

Bilateral total knee arthroplasty (BTKA) is a standard surgical option for treating knee diseases such as osteoarthritis, osteonecrosis, and rheumatoid arthritis, mainly in middle-aged and older patients [1]. As the population ages, the demand for total knee arthroplasty (TKA) continues to rise [2], with projections estimating about 935,000 annual procedures in the United States by 2030 [3]. Bilateral knee involvement is frequent in osteoarthritis [4, 5], and 36–46% of patients later undergo contralateral TKA [1]. Simultaneous bilateral TKA (SB-TKA) offers an efficient option, accounting for 4.0–5.6% of all TKA cases in the United States [6]. Compared to staged unilateral replacement, the bilateral approach offers several clear advantages, including shorter total recovery time and lower costs. However, it also creates several difficulties in perioperative care [7]. Acute postoperative pain remains one of the main concerns. The pain is often stronger and more persistent in bilateral TKA due to the extent of surgical trauma, which may slow down early mobilization and delay rehabilitation [8]. Postoperative nausea and vomiting (PONV) are also common adverse events following TKA. Their occurrence is affected by anesthetic drugs, opioid use, and patient-specific risk factors, and they can further postpone recovery and extend the length of hospital stay [9]. Glycemic management is another crucial element, particularly in patients with diabetes, since perioperative hyperglycemia has been associated with higher infection rates, wound problems, and poorer surgical outcomes [10].

The use of perioperative glucocorticoids has gained attention as a potential strategy to address these challenges. Evidence suggests that glucocorticoids may help reduce acute pain, suppress inflammation, and lower the risk of PONV, thereby enhancing functional recovery in bilateral TKA [11]. Prior studies, primarily in unilateral TKA, have demonstrated that perioperative glucocorticoids can significantly reduce postoperative pain and inflammation after TKA, with lower resting visual analog scale (VAS) scores on postoperative days 1 and 2, decreased opioid consumption by 2.89 mg morphine within 24 h, reduced levels of inflammatory markers including C-reactive protein (CRP) and interleukin-6 (IL-6), and reduced incidence of PONV [12–14]. Additionally, they have shown opioid-sparing effects and improved early functional recovery, such as a better range of motion (ROM) and ambulation. However, potential risks include transient hyperglycemia, particularly in diabetic patients, although overall complication rates, including infection and wound healing issues, appear unchanged in meta-analyses [15]. Glucocorticoids can be administered through multiple routes. Systemic methods include intravenous (IV) and oral dosing, while local techniques involve intra-articular injection, periarticular infiltration, or local infiltration analgesia. Each method presents different pharmacokinetic properties and potential benefits in symptom management. Nevertheless, their impact on perioperative glucose control raises concern, especially in vulnerable groups, and the optimal route to achieve a balance between safety and efficacy remains undefined.

Previous meta-analyses have primarily focused on unilateral TKA or mixed orthopedic populations, without specific emphasis on bilateral procedures [13, 15]. Although randomized controlled trials [RCTs] have provided valuable evidence, there is still no comprehensive review that has specifically assessed the effect of perioperative glucocorticoids on pain, PONV, and glycemic control in primary bilateral TKA. Clinical variability is expected among trials due to differences in glucocorticoid administration routes (intravenous vs. local infiltration), surgical settings (simultaneous vs. staged bilateral TKA), and trial designs (paired vs. independent knee procedures). Subgroup analyses were pre-specified to account for this heterogeneity. This systematic review and meta-analysis aim to fill the gap by synthesizing available RCT data and providing evidence-based guidance for clinical decision-making.

## Methods

### Search strategy

This systematic review and meta-analysis were conducted in accordance with the Preferred Reporting Items for Systematic Reviews and Meta-Analyses (PRISMA) guidelines [16]. The protocol adhered to the Cochrane Handbook for Systematic Reviews of Interventions and was prospectively registered on PROSPERO (Registration number: CRD420251152310). Three databases—PubMed, Web of Science, and Scopus—were searched from their inception to August 13, 2025, with no time restrictions applied to ensure comprehensive coverage of the relevant literature. The search strategy combined Medical Subject Headings (MeSH) and free-text keywords related to bilateral total knee arthroplasty and glucocorticoids. The full search strings, including Boolean operators, are provided in Table S1 within the supplementary materials. No grey literature or trial registry searches were conducted, as the focus was on English-language peer-reviewed randomized controlled trials (RCTs) to ensure high-quality evidence. However, reference lists of eligible papers and relevant reviews were screened for additional records. The search retrieved 1122 records from PubMed, 1033 from Web of Science, and 2708 from Scopus, totaling 4863 records across all databases. After removing 1028 duplicates, 3835 records underwent title and abstract screening, during which 3811 were excluded. A full-text review of 24 studies resulted in the inclusion of 5 trials.

### Inclusion and exclusion criteria

Eligible studies were randomized controlled trials (RCTs), published as full-text articles in peer-reviewed journals. The population consisted of adult patients (≥ 18 years) undergoing primary staged or simultaneous bilateral total knee arthroplasty under either general or regional anesthesia, regardless of indication [e.g., osteoarthritis, rheumatoid arthritis]. The intervention involved perioperative glucocorticoids, administered either locally (including local infiltration analgesia [LIA]) or systemically, standard within 24 h preoperatively, intraoperatively, or on the day of surgery. The choice of glucocorticoids and dosing windows was based on their established pharmacokinetic profiles, with systemic administration selected for rapid onset and systemic anti-inflammatory effects, and local administration (including LIA) for targeted pain relief and reduced systemic exposure, aligning with standard perioperative practices to optimize efficacy and safety. This included studies with combined interventions, such as glucocorticoids plus regional blocks, to evaluate their combined impact. Comparators were either placebo, no glucocorticoid, or standard care without steroids. Studies were required to report at least one of the following primary outcomes: Glycemia (post-anesthesia care unit (PACU), and postoperative day 1 (POD1), Postoperative nausea and vomiting (PONV) [incidence, severity measured by visual analog or PONV scales], Acute pain outcomes [pain scores at rest or with movement using VAS, or opioid/analgesic use expressed as morphine equivalents within (POD1–3)], Functional recovery [range of motion, patient-reported scores such as the Knee Society Score]. Secondary outcomes included inflammatory response (IL-6), hospital resource use [length of stay (LOS), readmission], and adverse events [infection, wound complications, pruritus, or bleeding]. Exclusion criteria were non-RCT designs, unilateral or revision arthroplasty, other joint procedures [e.g., hip], absence of primary outcome data, pediatric populations, and animal studies. For mixed total hip arthroplasty (THA)/total knee arthroplasty (TKA) trials without separable TKA data, studies were also excluded. Two reviewers independently screened records using Rayyan, resolving disagreements by discussion or arbitration with a third reviewer.

### Data extraction

Data were extracted independently by two reviewers using a predefined form. Extracted details covered study design, sample size, funding, patient demographics (age, sex, prevalence of diabetes), intervention (drug, dose, route, timing), comparator, and outcomes (means, SDs, risk ratios with 95% CIs). Continuous data were recorded as means and SDs where available; medians with interquartile ranges were converted to means and SDs using validated methods when necessary. Authors were contacted for missing or unclear data. Differences between reviewers were resolved by consensus. Pre-specified subgroup analyses were planned for: route of administration (intravenous [IV] vs. local/local infiltration analgesia [LIA]). However, we were unable to conduct a subgroup analysis for the surgical setting (simultaneous vs. staged bilateral TKA) due to the limited number of included trials. For outcome hierarchy and timing, primary time points were defined as POD 1–3 for pain and PACU/POD1 for glycemia. When multiple pain measures were reported, we prioritized pain with movement over rest, and peak scores over daily means. For glycemia, peak values were prioritized over means. If timings overlapped or were unclear, we used the closest available data point and noted this in the results.

### Quality assessment

The Cochrane Risk of Bias 2 (RoB 2) tool [17] was applied to evaluate study quality. Two reviewers assessed randomization, deviations from intended interventions, missing outcome data, outcome measurement, and selective reporting. Each domain was rated low risk, some concerns, or high risk of bias. Any disagreements were resolved by consensus. The certainty of evidence for the outcomes (pain, nausea, vomiting, and glycemia) was assessed using the Grading of Recommendations Assessment, Development and Evaluation (GRADE) framework, considering risk of bias, inconsistency, indirectness, imprecision, and publication bias [18] (Tables 4 and 5).

### Statistical analysis

All statistical analyses were performed using Stata version 17.0 (StataCorp LLC, College Station, TX, USA). Continuous outcomes were synthesized as mean differences (MDs) or standardized mean differences (SMDs), along with 95% confidence intervals (CIs). Dichotomous outcomes were expressed as risk ratios (RRs) with accompanying 95% CIs. A random-effects model based on the DerSimonian–Laird method was applied to account for between-study variability. Heterogeneity was assessed using the Cochran Q test (*p* < 0.10 considered significant) and quantified with the I^2^ statistic, with thresholds of 25%, 50%, and 75% indicating low, moderate, and high heterogeneity, respectively. When heterogeneity exceeded 50%, we conducted leave-one-out sensitivity analyses to evaluate the influence of individual studies on pooled estimates, and, when appropriate, outcomes were re-estimated using SMDs to address variability in measurement methods. Subgroup analyses were conducted based on the route of glucocorticoid administration (intravenous [IV] vs. local), and interaction p-values were reported for between-group differences. Assessment of publication bias was not feasible, as none of the outcomes included 10 or more studies, precluding a reliable evaluation with funnel plots or statistical tests. All analyses were two-tailed, and *p* < 0.05 was considered statistically significant. For studies involving bilateral total knee arthroplasty (BTKA), the patient was treated as the unit of analysis to avoid unit-of-analysis errors, in accordance with Cochrane guidance. Among the included RCTs, three trials (Chan et al. [25]; Kwon et al. [20]; Peng et al. [21]) used a paired-knee design, in which both knees of the same patient were assigned to different interventions. Chan et al. [25] accounted for within-patient correlation using paired statistical tests (paired t-test/Wilcoxon signed-rank), whereas Kwon et al. [20] and Peng et al. [21] reported unpaired analyses. For these trials, data were conservatively treated as independent in the primary meta-analysis, and potential correlation effects were explored through sensitivity analysis using an imputed intraclass correlation coefficient (ICC = 0.5). The remaining trials Jules-Elysee et al. [23]; Jules-Elysee et al. [19] employed a parallel-group design in which both knees received the same intervention; thus, no within-patient correlation adjustment was required.

## Results

### Search and screening

**Fig. 1:**
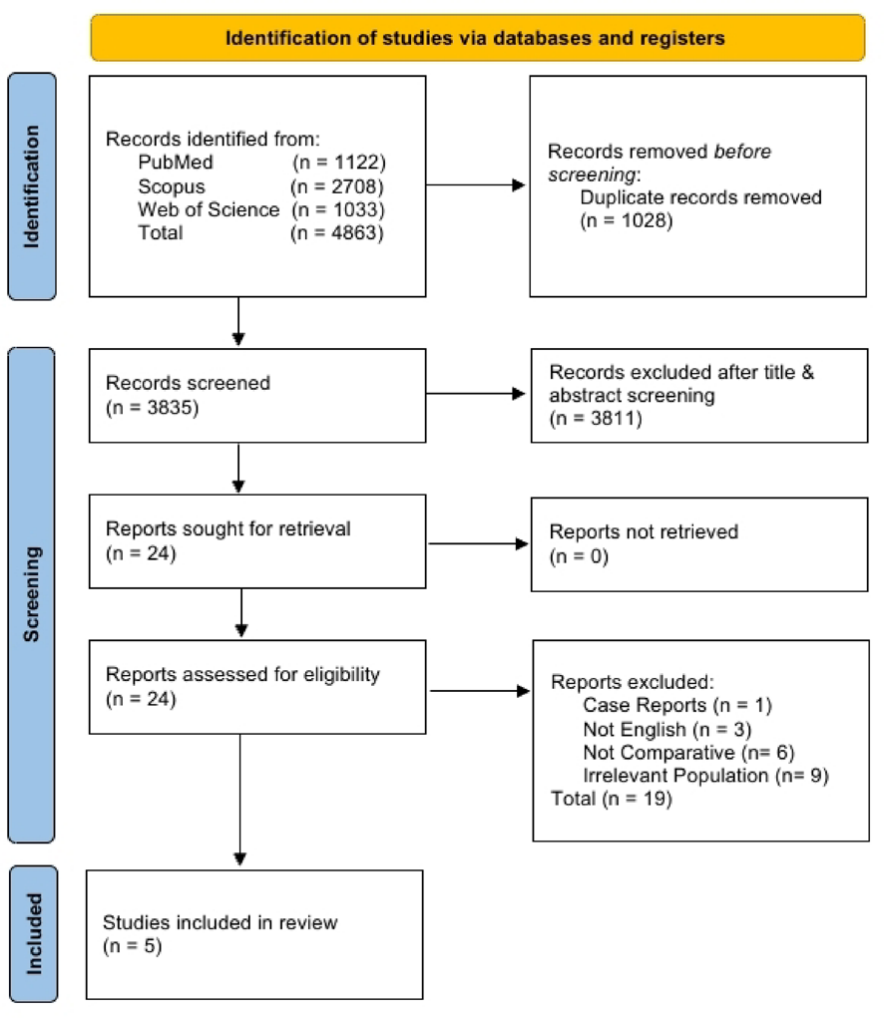
PRISMA flowchart of the selection process

A total of 4863 records were identified through database searching, including 1122 from PubMed, 1033 from Web of Science, and 2708 from Scopus. After the removal of 1028 duplicate records, 3,835 unique titles and abstracts were screened for eligibility. Of these, 3811 were excluded based on title and abstract screening. The remaining 24 articles were retrieved for full-text assessment, of which 19 were excluded because they didn’t meet the inclusion criteria. Finally, five studies [19–23] fulfilled all eligibility requirements and were included in the systematic review and subsequent analysis. The PRISMA flow diagram summarizes the selection process, as shown in Fig. 1.

### Study characteristics

Five randomized controlled trials (RCTs) [19–23] met the inclusion criteria and were included in the review, comprising a total of 245 participants. The trials were conducted in the USA (*n* = 2), China (*n* = 1), South Korea (*n* = 1), and China/Hong Kong (*n* = 1) between 2011 and 2021. All studies employed a parallel-group randomized design and involved patients undergoing total knee arthroplasty (TKA). Sample sizes ranged from 15 to 76 participants per study arm. The mean age of participants ranged from 64 to 71 years, with a predominance of female participants in most trials, as two studies specifically included only females. Reported body mass index (BMI) values generally fell within the overweight range (25–28 kg/m^2^). American Society of Anesthesiologists (ASA) grades I–III were most commonly represented, with high-risk patients (ASA ≥ 3 or uncontrolled comorbidities) systematically excluded across all studies. Duration of surgery or anesthesia ranged from approximately 68 min to 121 min. A detailed summary of study-level characteristics is provided in Table 1 (study design and intervention details) and Table 2 (demographic and perioperative variables).

**Table 1:** Summary of included studies

Study ID (Author, Year)	Country	Design	Sample size (*n*)	Type of arthroplasty	Anesthesia	Diabetes status (*n*)	Outcome measures
Jules-Elyseeet al. 2012	USA	RCT	34	Simultaneous BTKA	Spinal-epidural	2	IL-6, urinary desmosine, pain (VAS), fever, ROM, glucose, wound healing, complications
Jules-Elysee et al. 2011	USA	RCT	30	Simultaneous BTKA	Spinal + epidural + local	4	IL-6, hemodynamics, pain, ROM, glucose, complications, satisfaction
Peng et al. 2021	China	RCT	60	Simultaneous BTKA	Local	–	VAS pain, ROM, HSS score, swelling, drainage, complications, preference
Kwon et al. [20]	South Korea	RCT	76	Staged BTKA	Spinal	–	Night pain, ROM, SLR, AKS/WOMAC, satisfaction, complications
Chan et al. 2020	China	RCT	45	Simultaneous BTKA	Spinal + epidural	–	VAS pain, ROM, SLR, Knee Society Score, complications

**Table 2:** Baseline characteristics of include studies

Study	Age (Mean ± SD)	Male:Female (*n*)	BMI (Mean ± SD)	ASA grade	Comorbidities	Preop functional score (Mean ± SD)	Duration of surgery/anesthesia (Mean ± SD)
Jules-Elysee et al. 2012	–	–	–	≤ 2 (17)	Diabetes: 2/17	–	Intervention: 109.7 (16.5) Control: 116.0 (20.4)
Jules-Elysee et al. 2011	Intervention: 64 (7) Control: 71 (9)	Intervention: 6:9 Control: 9:6	–	Study group (12) Control (12)	–	–	Intervention: 121 (18) Control: 113 (15)
Peng et al. 2021	Intervention: 65.1 (6.8) Control: 65.1 (6.8)	Intervention: 4:56 Control: 4:56	Intervention: 27.9 (3.47) Control: 27.9 (3.47)	–	–	Intervention: 35.4 (3.6) Control: 36.0 (3.2)	–
Kwon et al. [20]	Intervention: 69.3 (3.15) Control: 69.3 (3.15)	Intervention: 0:76 Control: 0:76	Intervention: 26.45 (2.083) Control: 26.45 (2.083)	–	–	–	–
Chan et al. 2020	Intervention: 66 (5.8) Control: 66 (5.8)	Intervention: 14:31 Control: 14:31	Intervention: 28 (4.3) Control: 28 (4.3)	–	–	Intervention: KSS 43 (14) Control: KSS 42 (15)	Intervention: 72 (27) Control: 68 (23)

### Interventional characteristics

A variety of perioperative corticosteroid therapies administered via intravenous (IV) or local infiltration routes were examined in the five included randomized controlled studies [19–23]. Two early trials from the United States [19, 23] assessed systemic hydrocortisone administered intravenously at multiple perioperative time points. However, three Asian trials [20–22] investigated local steroid infiltration methods with betamethasone or triamcinolone administered periarticularly and, in one experiment, subcutaneously. Multiple 100 mg doses of hydrocortisone over the course of 24 h postoperatively were used in the systemic studies, while triamcinolone or betamethasone were employed in local infiltration at the time of wound closure or cement fixation. Multimodal local infiltration analgesia (LIA) mixtures free of corticosteroids or placebo saline were typically used as comparator arms. The anesthesia management techniques utilized in the studies also differed; all studies employed regional techniques (spinal ± epidural). Tourniquet use was not always reported; in certain instances, pressures as high as 250 mmHg were applied [21]. Table 3 provides a comprehensive comparison of the interventional features among the included trials.

**Table 3:** Interventional characteristics of the included studies

Study (Author, Year)	Country	Steroid (type, route, dose)	Timing / frequency	Comparator	Anesthesia	Tourniquet	Follow up	Key notes
Jules Elysee et al. 2012	USA	Hydrocortisone, IV, 100 mg ×3 doses	2 h preop, 8 h, 16 h postop (multiple)	Placebo (IV saline)	Combined spinal epidural	Yes (sequential)	6 months	Excluded high risk: ≥80 yrs, ASA ≥ 3, cardio-respiratory disease, DM HbA1c > 7%
Jules Elysee et al. 2011	USA	Hydrocortisone, IV, 100 mg ×2 doses	1st: 2 h preop, 2nd: 8 h postop	Placebo (IV saline, same schedule)	Spinal + epidural + local	Yes (both knees)	3 months	No NSAIDs allowed; 4 pts diabetic
Peng et al. 2021	China	Betamethasone periarticular (part of cocktail, 7 mg/mL)	Intraoperative, wound closure (local infiltration)	Cocktail without steroid (ropivacaine, flurbiprofen, TXA, morphine + saline)	Spinal + local	Yes, 250 mmHg	3 months	Cocktail enriched with multimodal analgesics; both unilateral/bilateral TKA
Kwon et al. [20]	South Korea	Triamcinolone acetonide periarticular, 40 mg	Intraop (before cement fixation & after polyethylene insertion)	Multimodal periarticular cocktail (morphine, ropivacaine, ketorolac, adrenaline; no steroid)	Spinal	NR	6 months	Female only, staged bilateral TKA (3 month interval)
Chan et al. 2020	China	Triamcinolone acetonide, periarticular + subcutaneous	Intraoperative, single dose	LIA cocktail (ropivacaine, ketorolac, adrenaline; no steroid)	Spinal + epidural	NR	12 months	Excluded uncontrolled DM, RA, immunodeficiency, prior infection

### Risk of bias assessment

**Fig. 2:**
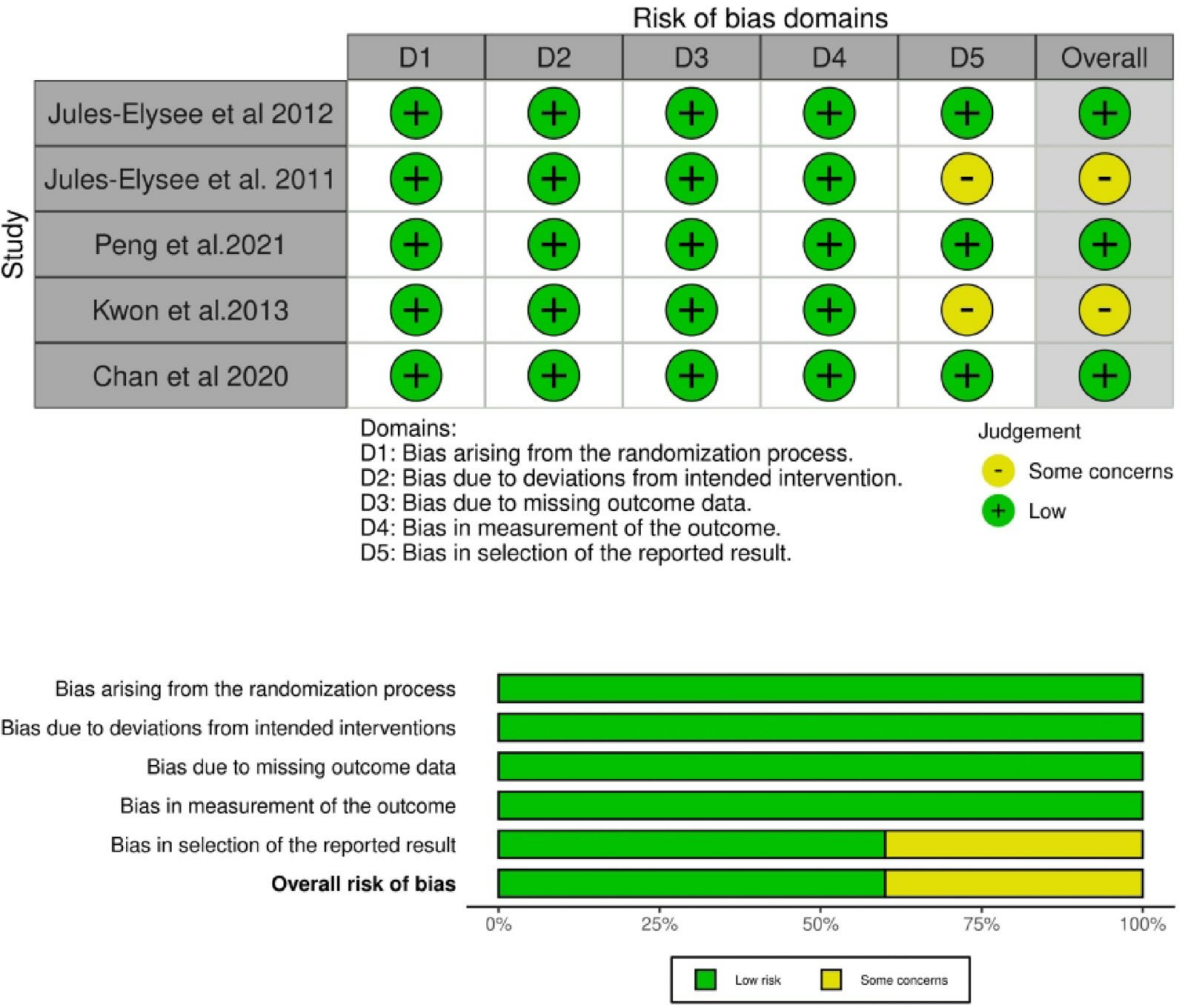
ROB assessment of the included RCTs

The Cochrane Risk of Bias 2 (RoB 2) tool was used to evaluate the methodological quality of the five included randomized controlled trials. This evaluation covered the following domains: (1) the randomization process, (2) deviations from intended interventions, (3) missing outcome data, (4) measurement of the outcome, and (5) selection of the reported result. Overall, three studies [19, 20, 22] demonstrated a low risk of bias across all domains. Two studies [21, 23] were rated as having “some concerns,” primarily due to issues in Domain 5 (Selection of the reported result), where insufficient details were available regarding pre-specified analysis plans or trial protocols to confidently confirm that all reported outcomes were pre-defined. No studies were judged to have a high risk of bias. A summary of the risk of bias assessment is presented in Fig. 2. The GRADE evaluation strengthened the reliability of our findings by assessing the certainty of the evidence. It assigned high certainty to postoperative glucose levels in the PACU and to the incidence of vomiting, while assigning moderate certainty to POD1 pain at rest and nausea, primarily due to the small number of contributing RCTs.

### Paired-knee handling

In the five randomized controlled trials (RCTs) included [19–23] on bilateral total knee arthroplasty (BTKA), the trial designs properly accounted for within-patient correlation. Three of these studies Chan et al. [25]; Kwon et al. [20]; Peng et al. [21] employed paired-knee designs. Among these, Chan et al. [25] used paired analyses to address within-patient correlation, whereas Kwon et al. [20] and Peng et al. [21] evaluated outcomes with unpaired methods. For the latter two studies, we performed sensitivity analysis using an imputed intraclass correlation coefficient (ICC = 0.5) to approximate potential within-patient correlation. Given that several outcomes included only a limited number of trials, such sensitivity analyses were conducted when feasible. These analyses did not materially alter the pooled estimates, confirming the robustness of the results. The remaining two trials Jules-Elysee et al. [23]; Jules-Elysee et al. [19] were parallel-group RCTs in which both knees within each patient received the same treatment; thus, the patient represented the analytical unit and no correlation adjustment was required. In general, using per-patient outcome data in all trials reduced unit-of-analysis bias and guaranteed a valid synthesis of the BTKA evidence base.

### Outcomes

#### Primary outcomes

##### Glycemic control

**Fig. 3:**
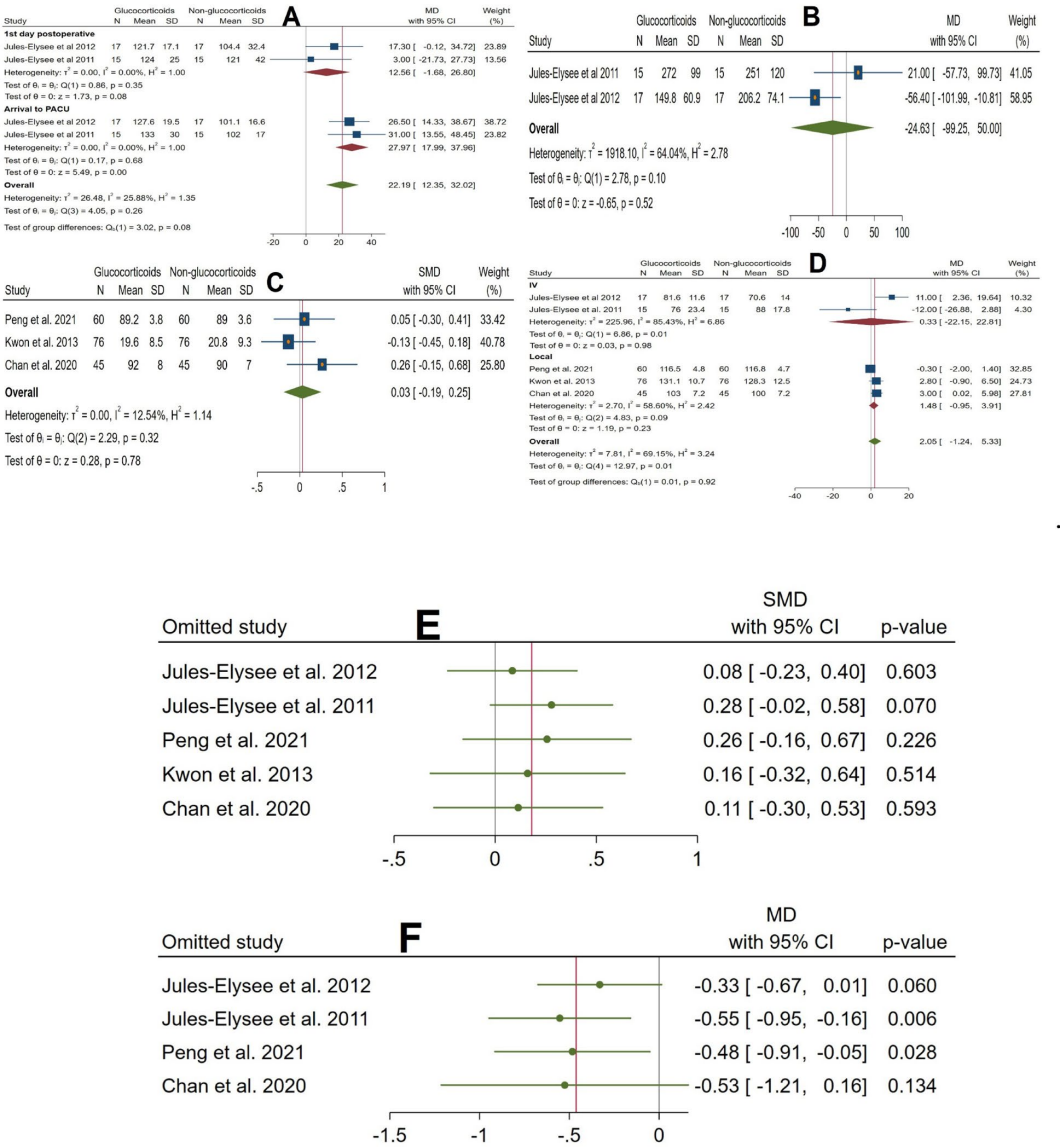

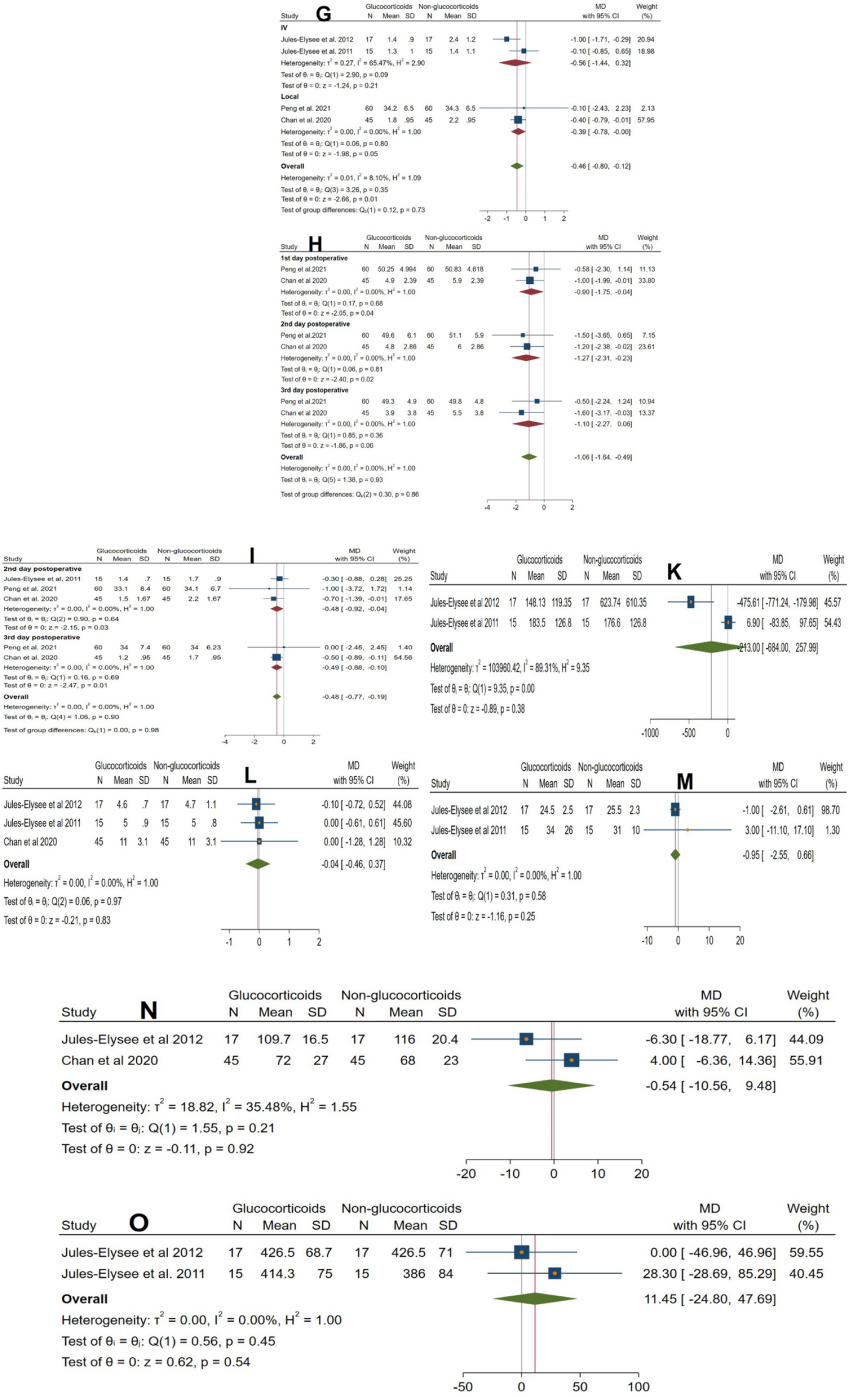

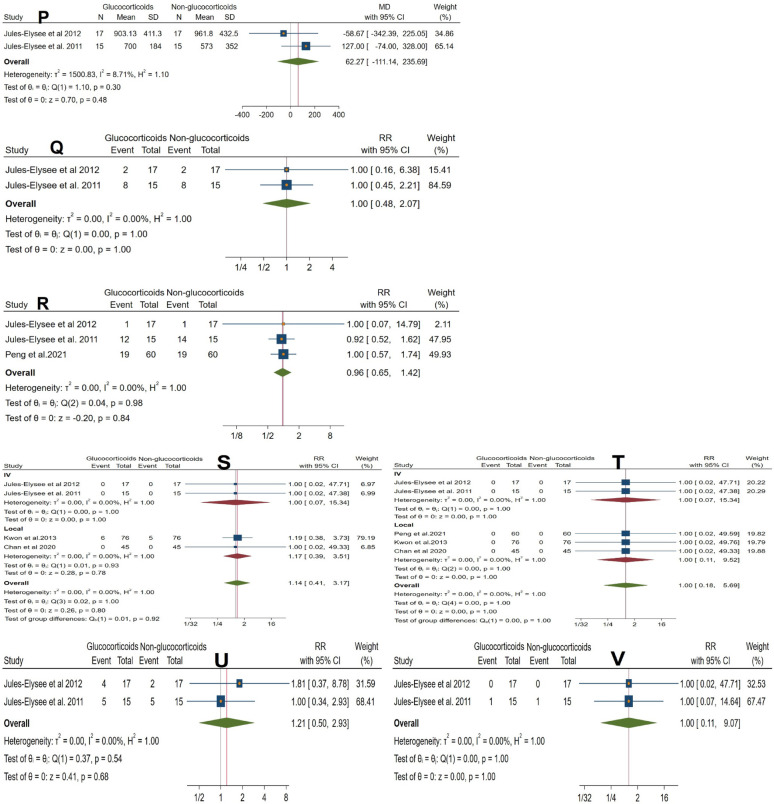
**A** Forest plot showing the difference in postoperative glucose levels between glucocorticoid and control groups. **B** Forest plot showing the difference in total opioid and analgesic consumption between groups. **C** Forest plot showing the difference in postoperative functional scores between groups. **D** Forest plot showing the difference in postoperative range of motion (degrees) between groups (forest plot). **E** Forest plot showing the difference in postoperative range of motion between groups (sensitivity analysis). **F** Forest plot showing the difference in VAS pain scores at rest on postoperative day 1 (sensitivity analysis). **G** Forest plot showing the difference in VAS pain scores at rest on postoperative day 1 between groups. **H** Forest plot showing the difference in VAS pain scores during activity between groups. **I** Forest plot showing the difference in VAS pain scores at rest on postoperative days 2 and 3 between groups. **K** Forest plot showing the difference in postoperative interleukin-6 (IL-6) levels between groups. **L** Forest plot showing the difference in length of hospital stay (days) between groups. **M** Forest plot showing the difference in PACU stay duration (hours) between groups. **N** Forest plot showing the difference in operative time (minutes) between groups. **O** Forest plot showing the difference in estimated blood loss (mL) between groups. **P** Forest plot showing the difference in blood transfusion volume (mL) within the first 24 h between groups. **Q** Forest plot showing the difference in the incidence of postoperative nausea between groups. **R** Forest plot showing the difference in the incidence of postoperative vomiting between groups. **S** Forest plot showing the difference in the incidence of wound complications between groups. **T** Forest plot showing the difference in the incidence of postoperative infection between groups. **U** Forest plot showing the difference in the incidence of postoperative pruritus between groups. **V** Forest plot showing the difference in the rate of readmission to the PACU between groups

Postoperative Glucose Levels were extracted from 2 RCTs [19, 23]. The pooled analysis revealed varying impacts on arrival at the post-anesthesia care unit (PACU) and the first postoperative day (POD1). At arrival to PACU, the mean difference (MD) was 27.97 (95% CI, 17.99 to 37.96; *p* = 0.00), indicating a statistically significant increase in glucose levels. On POD1, the MD was 12.56 (95% CI, − 1.68 to 26.80; *p* = 0.08), which was not statistically significant. Overall, the pooled MD across both time points was 22.19 (95% CI, 12.35 to 32.02; *p* < 0.001), indicating a statistically significant increase in glucose levels. Heterogeneity was moderate (I^2^ = 25.88% overall, I^2^ = 0% for each subgroup), and subgroup differences were not significant (*p* = 0.08), suggesting a consistent effect of glucocorticoids on elevating glucose levels from arrival to PACU through POD1. This analysis included studies utilizing only intravenous (IV) glucocorticoid administration (Fig. 3A).

##### Opioid and analgesic consumption

Two RCTs [19, 23] provided data on opioid and analgesic consumption. The pooled analysis revealed a reduction in opioid and analgesic use in the glucocorticoid group; however, this reduction was not statistically significant [MD = − 24.63 mg, 95% CI − 99.25 to 50.00, *p* = 0.52]. With moderate heterogeneity (I^2^ = 64.04%). (Fig. 3B) This analysis included studies utilizing only intravenous glucocorticoid administration.

##### Functional recovery

The pooled analysis indicated no statistically significant difference in postoperative functional scores among three RCTs [20–22] between the glucocorticoid and non-glucocorticoid groups [SMD = 0.03, 95% CI − 0.19 to 0.25, *p* = 0.78]. Heterogeneity was low (I^2^ = 12.54%), and the analysis included studies utilizing only local glucocorticoid administration. (Fig. 3C) Five RCTs [19–23] reported data on postoperative range of motion. The pooled analysis showed that the administration of glucocorticoids did not result in a statistically significant difference in postoperative range of motion [MD = 2.05, 95% CI − 1.24 to 5.33, *p* = 0.23], with moderate heterogeneity (I^2^ = 69.15%). The intravenous (IV) subgroup showed an MD of 0.33 (95% CI − 22.15 to 22.81; *p* = 0.98), which was not statistically significant, with high heterogeneity (I^2^ = 85.43%). The local subgroup demonstrated an MD of 1.48 (95% CI − 0.95 to 3.91; *p* = 0.23), also not statistically significant, with moderate heterogeneity (I^2^ = 58.60%). (Fig. 3D) Sensitivity analysis for range of motion was conducted and indicated the robustness of the original analysis, as no single study substantially influenced the overall non-significant result for ROM (Fig. 3E).

##### Pain management

Four RCTs [19, 21–23] provided data on VAS pain score. The pooled analysis revealed that glucocorticoid use led to a statistically significant reduction in VAS pain scores at rest on the first postoperative day [MD = − 0.46, 95% CI − 0.80 to − 0.12, *p* = 0.01]. A subgroup analysis based on the route of administration [Intravenous vs. local] showed no statistically significant difference in treatment effect [*p* = 0.73 for subgroup differences], indicating consistent analgesic efficacy across different administration methods. The intravenous (IV) subgroup showed a mean difference (MD) of − 0.56 (95% CI, − 1.44 to 0.32; *p* = 0.21), which was not statistically significant. In contrast, the local subgroup demonstrated a statistically significant MD of − 0.39 (95% CI, − 0.78 to − 0.00; *p* = 0.05). (Fig. 3G) Sensitivity analysis confirmed the robustness of this finding. (Fig. 3F) Pain at rest remained significantly reduced on the second and the third postoperative days, with an MD of − 0.48 (95% CI, − 0.77 to − 0.19; *p* = 0.05) [11–13]. Notably, the data for the third postoperative day were exclusively derived from studies utilizing local glucocorticoids. (Fig. 3I)

Pain during activity was reported by two RCTs [21, 22] and analyzed exclusively in studies using local administration. The pooled analysis revealed significant reductions in pain across postoperative days (POD) 1 and 2, with POD 1 showing (MD) of − 0.90 (95% CI, − 1.75 to − 0.04; *p* = 0.04) and POD 2 demonstrating a statistically significant MD of − 1.27 (95% CI, − 2.31 to − 0.23; *p* = 0.02). However, the reduction on POD 3 was not statistically significant, with an MD of − 1.10 (95% CI, − 2.27 to 0.06; *p* = 0.06). The overall pooled mean difference across all time points was − 1.06 (95% CI, − 1.64 to − 0.49; *p* < 0.001), indicating a statistically significant reduction in VAS scores during activity. Heterogeneity remained low (I^2^ = 0%), and subgroup differences between postoperative days were not significant (*p* = 0.86) (Fig. 3H).

##### Postoperative nausea and vomiting (PONV)

Glucocorticoid use had no significant effect on postoperative nausea or vomiting. The pooled analysis revealed nausea (RR = 1.00, 95% CI 0.48 to 2.07, *p* = 1.00; I^2^ = 0%) and vomiting (RR = 0.96, 95% CI 0.65 to 1.42, *p* = 0.84; I^2^ = 0%). Nausea was assessed in two RCTs [19, 23] using only intravenous (IV) glucocorticoid administration, while vomiting was reported in three RCTs [19, 21, 23], including both IV and local routes (Fig. 3Q and R).

#### Secondary outcomes

No significant effects were observed across secondary outcomes. Glucocorticoids did not reduce inflammatory response (IL-6: MD = − 213.00 pg/mL, 95% CI − 684.00 to 257.99; *p* = 0.38; I^2^ = 89%; Fig. 3K) or affect resource utilization, including length of hospital stay (LOS: MD = − 0.04 days, 95% CI − 0.46 to 0.37; *p* = 0.83; I^2^ = 0%; Fig. 3L), PACU stay (MD = − 0.95 h, 95% CI − 2.55 to 0.66; *p* = 0.25; I^2^ = 0%; Fig. 3M), or operative parameters such as estimated blood loss (MD = 11.45 mL, 95% CI − 24.80 to 47.69; *p* = 0.54; I^2^ = 0%; Fig. 3O), blood transfusion volume (MD = 62.27 mL, 95% CI − 111.14 to 235.69; *p* = 0.48; I^2^ = 0%; Fig. 3P), or operative time (MD = − 0.54 min, 95% CI − 10.56 to 9.48; *p* = 0.92; Fig. 3N). Safety outcomes remained unaffected, with no increase in wound complications (RR = 1.14, 95% CI 0.41 to 3.17; *p* = 0.80; I^2^ = 0%; Fig. 3S), infection rates (RR = 1.00, 95% CI 0.18 to 5.69; *p* = 1.00; I^2^ = 0%; Fig. 3T), pruritus (RR = 1.21, 95% CI 0.50 to 2.93; *p* = 0.68; Fig. 3U), or PACU readmission (RR = 1.00, 95% CI 0.11 to 9.07; *p* = 1.00; Fig. 3V). All analyses showed low to no heterogeneity except IL-6 (Tables 4 and 5).

**Table 4:** Summary of findings (SoF) table – GRADE assessment of certainty of evidence

Certainty assessment							No of patients		Effect		Certainty	Importance
№ of studies	Study design	Risk of bias	Inconsistency	Indirectness	Imprecision	Other considerations	[intervention]	[comparison]	Relative (95% CI)	Absolute (95% CI)		
VAS at rest 1st day												
4	Randomised trials	Not serious	Serious^a^	Not serious	Not serious	None	137	137	–	MD **0.46 lower** (0.8 lower to 0.12 lower)	⨁⨁⨁◯ Moderate^a^	CRITICAL
Postoperative glucose level 1st day												
2	Randomised trials	Not serious	Not serious	Not serious	serious^b^	None	32	32	–	MD **12.56 higher** (1.68 lower to 26.8 higher)	⨁⨁⨁◯ Moderate^b^	CRITICAL
Postoperative glucose level (PACU)												
2	Randomised trials	Not serious	Not serious	Not serious	not serious	None	32	32	–	**27.97 higher** (17.99 higher to 32.02 higher)	⨁⨁⨁⨁ High	CRITICAL
Postoperative Nausea												
2	Randomised trials	Not serious	Not serious	Not serious	serious^b^	None	10/32 (31.3%)	10/32 (31.3%)	**RR 1.00** (0.48 to 2.07)	**0 fewer per 1**,**000** (from 163 fewer to 334 more)	⨁⨁⨁◯ Moderate^b^	CRITICAL
Postoperative vomiting												
3	Randomised trials	Not serious	Not serious	Not serious	Not serious	None	32/92 (34.8%)	34/92 (37.0%)	**RR 0.96** (0.65 to 1.42)	**15 fewer per 1**,**000** (from 129 fewer to 155 more)	⨁⨁⨁⨁ High	CRITICAL

**Table 5:** Summary of findings: effect of perioperative glucocorticoids on postoperative outcomes

Outcomes	Anticipated absolute effects*(95% CI)		Relative effect (95% CI)	No of participants (studies)	Certainty of the evidence (GRADE)	Comments
	Risk with [comparison]	Risk with [intervention]				
VAS at rest 1st day	The mean VAS at rest 1st day was **0**	MD **0.46 lower** (0.8 lower to 0.12 lower)	–	274 (4 RCTs)	⨁⨁⨁◯ Moderate^a^	Glucocorticoids likely results in a large reduction in VAS at rest 1st day.
Postoperative glucose level 1st day	The mean postoperative Glucose Level 1st day was **0**	MD **12.56 higher** (1.68 lower to 26.8 higher)	–	64 (2 RCTs)	⨁⨁⨁◯ Moderate^b^	Glucocorticoids probably does not increase postoperative Glucose Level 1st day.
Postoperative glucose level (PACU)	The mean Postoperative Glucose Level (PACU) was **0**	**27.97 higher** (17.99 higher to 32.02 higher)	–	64 (2 RCTs)	⨁⨁⨁⨁ High	Glucocorticoids results in large increase in Postoperative Glucose Level (PACU).
Postoperative nausea	313 per 1000	**313 per 1**,**000** (150 to 647)	**RR 1.00** (0.48 to 2.07)	64 (2 RCTs)	⨁⨁⨁◯ Moderate^b^	Glucocorticoids likely results in little to no difference in Postoperative Nausea.
Postoperative vomiting	370 per 1000	**355 per 1**,**000** (240 to 525)	**RR 0.96** (0.65 to 1.42)	184 (3 RCTs)	⨁⨁⨁⨁ High	Glucocorticoids results in little to no difference in Postoperative Vomiting.

## Discussion

Effective pain management after surgery, preventing nausea, and enhancing recovery are crucial for optimal outcomes in bilateral total knee arthroplasty (BTKA), which entails greater surgical stress than unilateral TKA. This systematic review and meta-analysis gathered data from five randomized controlled trials (RCTs) [19–23] to evaluate the effectiveness and safety of glucocorticoids during the perioperative phase in BTKA. Our findings indicate that glucocorticoids significantly reduce early postoperative pain, particularly when administered locally via the periarticular route, and when combined with local infiltration analgesia (LIA). However, they do not significantly affect postoperative nausea and vomiting (PONV), opioid consumption, or functional recovery. There is limited information on glycemic control, and the safety profile appears positive, showing no increased risk of complications.

Our analysis revealed a notable decrease in visual analog scale (VAS) pain scores at rest on the postoperative day 1 (POD1) (MD = − 0.46, 95% CI − 0.80 to − 0.12, *p* = 0.01, based on four RCTs). This improvement persisted into POD2 and POD3, particularly in trials that utilized betamethasone or triamcinolone locally [20–22]. The subgroup analysis showed a significant effect for local administration (MD = − 0.39, 95% CI − 0.78 to 0.00, *p* = 0.05), but not for intravenous (IV) administration (MD = − 0.56, 95% CI − 1.44 to 0.32, *p* = 0.21), indicating differences based on the administration method. Pain experienced during activity, assessed in two local administration trials [20, 21], showed notable decreases on POD1 (MD = − 0.90, 95% CI − 1.75 to − 0.04, *p* = 0.04) and POD2 (MD = − 1.27, 95% CI − 2.31 to − 0.23, *p* = 0.02), but not on POD3 (MD = − 1.10, 95% CI − 2.27 to 0.06, *p* = 0.06), indicating a temporary advantage. These results are consistent with research on unilateral TKA, which suggests that IV dexamethasone alleviates early pain [24, 25]. The reliability of pain reduction was supported by meta-analyses, which reported moderate yet statistically significant pain reductions with perioperative steroids [26, 27]. While these reductions (ranging from − 0.46 to − 1.27 mm on the VAS) are modest and fall below the commonly accepted 10–20 mm threshold for clinical significance, they may still contribute to tangible improvements in patient function, such as slight enhancements in mobility or reduced stiffness, and a modest decrease in opioid consumption by alleviating acute postoperative pain, particularly in the early postoperative period. The clinical impact, however, may vary depending on individual patient responses and the consistency of these effects across the four RCTs. Although local trials yielded mixed outcomes, possibly due to variations in local administration or surgical methods, like the use of tourniquets [21], IV hydrocortisone studies [19, 23] suggested early pain relief, likely due to its systemic anti-inflammatory effects. This suggests that in the high-inflammatory context of BTKA, IV glucocorticoids may provide more consistent systemic pain relief. At the same time, the success of local administration may depend on procedural factors, underscoring the need for further research to improve local techniques.

No notable effects were found for PONV or opioid use. Combined results from two RCTs for nausea (RR = 1.00, 95% CI 0.48 to 2.07, *p* = 1.00) and three RCTs for vomiting (RR = 0.96, 95% CI 0.65 to 1.42, *p* = 0.84) indicated no advantage, with no heterogeneity (I^2^ = 0%). Opioid use (two RCTs) also did not show a significant decrease (MD = -24.63 mg, 95% CI -99.25 to 50.00, *p* = 0.52, I^2^ = 64.04%). These findings contrast with studies on unilateral TKA that indicated antiemetic and opioid-sparing benefits [24–27]. This discrepancy is likely due to the limited number of BTKA-specific RCTs and significant variability in opioid data. Furthermore, differences in glucocorticoid dosage and timing, along with variations in the types of agents used (such as hydrocortisone and dexamethasone) and their administration methods (Intravenous versus local), may have contributed to the observed lack of PONV reduction. Additionally, the use of concurrent antiemetics in specific trials may have obscured the antiemetic effects of glucocorticoids, which could have contributed to this result. Since only BTKA-specific trials were included, excluding mixed THA/TKA trials as per our criteria, these findings suggest that glucocorticoids do not consistently lower PONV or opioid consumption in BTKA, and clinicians should not expect these benefits based on unilateral TKA studies [28, 29].

Data on glycemic control, based on two RCTs [19, 23], indicated a notable rise in glucose levels in the post-anesthesia care unit (PACU) (MD = 27.97, 95% CI 17.99 to 37.96, *p* < 0.001), but not on POD1 (MD = 12.56, 95% CI − 1.68 to 26.80, *p* = 0.08). The overall effect (MD = 22.19, 95% CI 12.35 to 32.02, *p* < 0.001) suggests temporary hyperglycemia, although the limited number of trials diminishes certainty. Previous research indicates that this effect peaks within 24 h and resolves by 48 h [29, 30], with no adverse outcomes associated with TKA [31, 32]. Nonetheless, the lack of specific data for BTKA prevents making conclusive statements about normalization by POD1; therefore, glucose monitoring is advised, especially for diabetic patients receiving IV glucocorticoids. Functional outcomes showed no significant improvement, with no differences in postoperative functional scores (SMD = 0.03, 95% CI − 0.19 to 0.25, *p* = 0.78, three RCTs) or ROM (MD = 2.05, 95% CI − 1.24 to 5.33, *p* = 0.23, five RCTs). The IV ROM subgroup exhibited high heterogeneity (I^2^ = 85.43%), partly due to one trial [19] that reported per-knee ROM data, for which only data from the right knee were used to avoid unit-of-analysis errors. Sensitivity analysis confirmed robustness. These findings of functional recovery align with prior reviews [26, 33]. The safety results were encouraging, showing no higher risk of wound complications (RR = 1.14, 95% CI 0.41 to 3.17, *p* = 0.80, based on four RCTs) or infections (RR = 1.00, 95% CI 0.18 to 5.69, *p* = 1.00, based on five RCTs). This is consistent with results from extensive cohort studies [34, 35]. Other safety outcomes, such as pruritus (RR = 1.21, 95% CI 0.50 to 2.93, *p* = 0.68) and PACU readmission (RR = 1.00, 95% CI 0.11 to 9.07, *p* = 1.00), did not reveal any significant differences. Inflammatory markers (IL-6) and resource utilization (length of stay, PACU stay) also showed no significant effects.

The clinical guidance based on our results suggests that local administration of betamethasone or triamcinolone significantly reduces pain at rest (POD1–3) and during activity (POD1–2) in simultaneous bilateral total knee arthroplasty (BTKA) [20–22]. However, its efficacy may vary depending on the surgical techniques used (e.g., tourniquet use). Intravenous (IV) administration of hydrocortisone or dexamethasone did not show significant pain reduction at rest and should not be relied upon for this purpose in BTKA. Data for staged BTKA are limited but suggest similar pain reduction at rest on POD1 with LIA. Multi-dose IV treatments [19, 23] did not provide extra benefits and could raise the risk of hyperglycemia, indicating that single-dose IV, if utilized, should be used with caution. Given the lack of postoperative nausea and vomiting (PONV) or opioid-sparing effects, glucocorticoids should not be used for these outcomes. Glucose monitoring is essential with IV administration due to transient hyperglycemia. Glucocorticoids, particularly through local administration, may complement multimodal pain control strategies within enhanced recovery protocols for simultaneous BTKA; however, current evidence does not yet support routine ERAS incorporation.

This systematic review has many strengths. It focuses solely on BTKA, offering a clinically relevant perspective for this high-risk group. By combining both IV (hydrocortisone, dexamethasone) and local (betamethasone, triamcinolone) methods, it improves applicability compared to reviews that only look at dexamethasone. The exclusive use of RCTs and the Cochrane RoB 2 tool guarantees methodological rigor, with most studies indicating low to moderate risk of bias. Our GRADE assessment enhances reliability by assessing evidence certainty, confirming high certainty for glycemia and postoperative vomiting, but moderate certainty for pain outcomes and postoperative nausea due to the limited number of trials. A significant strength of this meta-analysis is the careful management of paired-knee data across bilateral TKA trials. By evaluating outcomes per patient whenever feasible and conducting sensitivity analyses using an imputed intra-class correlation coefficient (ICC = 0.5) when relevant, we tackled potential within-patient correlation. This approach minimized unit-of-analysis errors and confirmed the robustness of pooled estimates even in studies that initially reported unpaired analyses. Furthermore, one trial that provided per-knee data was appropriately harmonized by using right-knee ROM values to ensure consistent patient-level synthesis. However, there are limitations, such as the small number of RCTs (*n* = 5), especially for glycemia (two RCTs), PONV (two to three RCTs), and opioid use (two RCTs), which restricts statistical power and certainty. Because several outcomes included only a limited number of trials, sensitivity analyses for within-patient correlation were conducted only when feasible, which may limit the generalizability of correlation-adjusted findings. Differences in glucocorticoid agents, dosages, and administration methods (e.g., single vs. multi-dose IV, local techniques) may influence generalizability. The considerable heterogeneity in IV ROM outcomes (I^2^ = 85.43%) partly reflects the treatment of per-knee data. Older trials may not align with current ERAS protocols, and the lack of long-term outcome data limits the conclusions about functional recovery. The differences in anesthesia types (general vs. regional), the use of tourniquets, and the methods of administering glucocorticoids (intravenous [IV] vs. local, including local infiltration analgesia [LIA]) in the included trials may limit the applicability of these results. For example, the success of local administration varied depending on procedural elements, such as the use of tourniquets. Older studies may not accurately represent the current Enhanced Recovery After Surgery (ERAS) protocols, which utilize standardized anesthesia techniques. Therefore, even though the results indicate benefits in reducing pain, their relevance to various BTKA protocols should be carefully considered, highlighting the need for further research to standardize these factors. Future studies should aim for larger, BTKA-specific randomized controlled trials (RCTs), employ appropriate paired analyses for within-patient designs, and directly compare IV versus local administration as well as simultaneous versus staged procedures to strengthen future clinical recommendations.

## Conclusion

In conclusion, perioperative glucocorticoids appear to reduce early postoperative pain in bilateral total knee arthroplasty (BTKA), particularly when administered through local infiltration analgesia (LIA), without evidence of increased complications. However, despite prior evidence suggesting postoperative nausea and vomiting (PONV) reduction in unilateral TKA, our findings showed no significant effect on PONV incidence in BTKA, nor on opioid consumption or functional recovery. These discrepancies likely reflect the small number of BTKA-specific randomized controlled trials and heterogeneity in perioperative protocols. Transient elevations in blood glucose were observed following intravenous (IV) administration, highlighting the importance of careful glucose monitoring, particularly in patients with diabetes. Clinicians may consider LIA as part of multimodal analgesia strategies for simultaneous BTKA to achieve short-term pain relief, while exercising caution with systemic IV use. Larger, well-designed BTKA-specific RCTs are needed to clarify the effects of glucocorticoids on PONV, glycemic outcomes, and recovery parameters before these agents can be considered a standard component of Enhanced Recovery After Surgery (ERAS) protocols.

## Supplementary Information

Below is the link to the electronic supplementary material.

Supplementary Material 1

## Data Availability

All data generated or analyzed during this study are included in the published article.

## Abbreviations

TKA: Total knee arthroplasty
BTKA: Bilateral total knee arthroplasty
PONV: Postoperative nausea and vomiting
VAS: Visual analog scale
IL-6: Interleukin-6
LOS: Length of stay
PACU: Post-anesthesia care unit
LIA: Local infiltration analgesia
SD: Standard deviation
SMD: Standardized mean difference
MD: Mean difference
CIs: Confidence intervals
RCT: Randomized controlled trial
POD1: Postoperative day 1
ERAS: Enhanced recovery after surgery
