# Supplementary material for: Impact of perioperative glucocorticoids on glycemic control, PONV, and acute pain after primary bilateral total knee arthroplasty: a systematic review and meta-analysis of randomized controlled trials
Source: J Orthop Surg Res. 2025 Dec 5;20:1066. doi: 10.1186/s13018-025-06521-5 (PMC12690901; doi:10.1186/s13018-025-06521-5)
Supplement: Supplementary file 1 — Supplementary Material 1 [file 13018_2025_6521_MOESM1_ESM.docx]

| **Database** | **Search Strings** | **Number of Outcomes** |
| --- | --- | --- |
| PubMed | (("total knee arthroplast*"[tiab] OR "total knee replacement"[tiab] OR "TKA"[tiab] OR "TKR"[tiab] OR "knee arthroplasty"[tiab] OR "knee replacement"[tiab] OR "bilateral total knee arthroplasty"[tiab] OR "primary bilateral total knee arthroplasty"[tiab] OR "simultaneous bilateral tka"[tiab] OR "bilateral knee replacement"[tiab] OR "bilateral tka"[tiab] OR "Arthroplasty, Replacement, Knee"[Mesh]) AND ("Glucocorticoid*"[Mesh] OR "Adrenal Cortex Hormones"[Mesh] OR "Cortisone"[Mesh] OR "Dexamethasone"[Mesh] OR "Betamethasone"[Mesh] OR "Methylprednisolone"[Mesh] OR "Hydrocortisone"[Mesh] OR "Triamcinolone"[Mesh] OR "Prednisone"[Mesh] OR "Prednisolone"[Mesh] OR "Cortisone"[tiab] OR "Dexamethasone"[tiab] OR "Betamethasone"[tiab] OR "Methylprednisolone"[tiab] OR "Hydrocortisone"[tiab] OR "Triamcinolone"[tiab] OR "Prednisone"[tiab] OR "Prednisolone"[tiab] OR "steroid*"[tiab] OR "corticosteroid*"[tiab])) | 1122 |
| Scopus | (("total knee arthroplast*" OR "total knee replacement" OR "TKA" OR "TKR" OR "knee arthroplasty" OR "knee replacement" OR "bilateral total knee arthroplasty" OR "primary bilateral total knee arthroplasty" OR "simultaneous bilateral tka" OR "bilateral knee replacement" OR "bilateral tka" OR "Arthroplasty, Replacement, Knee") AND ("Glucocorticoid*" OR "Adrenal Cortex Hormones" OR "Cortisone" OR "Dexamethasone" OR "Betamethasone" OR "Methylprednisolone" OR "Hydrocortisone" OR "Triamcinolone" OR "Prednisone" OR "Prednisolone" OR "steroid*" OR "corticosteroid*")) | 2708 |
| Web of Science (WOS) | (("total knee arthroplast*" OR "total knee replacement" OR "TKA" OR "TKR" OR "knee arthroplasty" OR "knee replacement" OR "bilateral total knee arthroplasty" OR "primary bilateral total knee arthroplasty" OR "simultaneous bilateral tka" OR "bilateral knee replacement" OR "bilateral tka" OR "Arthroplasty, Replacement, Knee") AND ("Glucocorticoid*" OR "Adrenal Cortex Hormones" OR "Cortisone" OR "Dexamethasone" OR "Betamethasone" OR "Methylprednisolone" OR "Hydrocortisone" OR "Triamcinolone" OR "Prednisone" OR "Prednisolone" OR "steroid*" OR "corticosteroid*")) | 1033 |

**Table S1: The full search strings, including Boolean operators**
